# Doxorubicin induces cardiotoxicity by enhancing autophagy via mTOR signaling in hiPSC- and hESC-derived cardiomyocytes

**DOI:** 10.3389/fcell.2025.1616235

**Published:** 2025-11-25

**Authors:** Minxia Ke, Hao Wang, Kailun Yang, Meng Ji, Nianmin Qi, Yuehong Wu

**Affiliations:** 1 Department of Biochemistry and Molecular Biology, College of Life Science and Medicine, Zhejiang Sci-Tech University, Hangzhou, Zhejiang, China; 2 Hangzhou Biaomo Biosciences Co., Ltd., Hangzhou, Zhejiang, China; 3 Asia Stem Cell Therapies Co., Limited, Shanghai, China

**Keywords:** doxorubicin, cardiomyocytes, autophagy, MTOR signaling, human induced pluripotent stem cells, human embryonic stem cells

## Abstract

**Introduction:**

Doxorubicin (DOX) is a highly effective anti-cancer drug, but its clinical applications are limited by its cardiotoxicity. The mechanisms underlying DOX-induced cardiotoxicity (DIC) remain incompletely understood. Human induced pluripotent stem cells (hiPSCs) and human embryonic stem cells (hESCs) offer an advanced platform for investigating DIC, as they accurately recapitulate human cardiac physiology and pathology. However, the roles and mechanisms of DIC in hiPSC-CMs and hESC-CMs, especially regarding autophagy dynamics and regulation, are still not well-defined.

**Methods:**

Cell viability, apoptosis, reactive oxygen species production, and DNA damage were assessed. Autophagy was evaluated by transmission electron microscope, LC3-II/LC3-I ratio, and autophagy flux assays. The role of autophagy and mTOR signaling was investigated using 3-methyladenine (3-MA) and rapamycin (RAPA), respectively.

**Results:**

DOX reduced cell viability and induced apoptosis in hiPSC-CMs and hESC-CMs. Additionally, DOX caused an increase in reactive oxygen species production and DNA damage. Furthermore, DOX significantly upregulated autophagy, confirmed by the accumulation of autophagosomes and autolysosomes, and an increase in the LC3-II/LC3-I ratio. Autophagy flux assays showed that DOX induced autophagy in a time-dependent manner. The autophagy mediated by DOX was partially attenuated by 3-MA. Moreover, this activation was due to mTOR signaling inhibition. The downregulation of mTOR signaling by RAPA increased cell death of hESC-CMs. Interestingly, minor variations in injury severity and cellular sensitivity were observed between these two models.

**Conclusion:**

Our study uncovered the multifaceted effects of DOX on hiPSC-CMs and hESC-CMs, revealing a shared mechanism in which DOX enhances autophagy via inhibition of the mTOR signaling pathway. These findings reveal key insights into DIC pathogenesis and suggest that autophagy modulation may be a promising therapeutic strategy.

## 1 Introduction

Doxorubicin (DOX) is an effective anti-cancer agent for treating a wide range of malignancies, but its cumulative and dose-dependent cardiotoxicity limits its clinical application (Abdullah et al., 2019; Jones and Dass, 2022). Despite decades of studies, the mechanisms underlying DOX-induced cardiotoxicity (DIC) have not been fully elucidated, and predicting or preventing DIC in individual patients remains challenging.

Several different molecular mechanisms have been proposed for DIC in both experimental and clinical studies. The dominant mechanism is associated with DNA damage and reactive oxygen species (ROS) production, which leads to cardiomyocyte death (Li et al., 2020; L'Ecuyer et al., 2006). Other hypotheses have been proposed, including mitochondrial dysfunction and the modulation of intracellular calcium release (Hanna et al., 2014; Ichikawa et al., 2014). In addition, autophagy has also been proposed to serve a dual role in DIC (Sun et al., 2023; Xiao et al., 2019). Autophagy, a multistep and dynamic biological process, plays a crucial role in maintaining cellular homeostasis (Wang et al., 2024). Many studies indicate that autophagy is involved in several physiological and pathological processes in the heart (Li et al., 2024; Yamaguchi, 2019). Several studies have shown that DOX suppresses basal autophagy in rat cardiomyocytes, suggesting that activation of autophagy may protect against DIC (Pizarro et al., 2016; Sishi et al., 2013; Zilinyi et al., 2018). In contrast, other studies report that DOX upregulates autophagy in rat cardiomyocytes, contributing to detrimental effects and cell death (Kobayashi et al., 2010; Scicchitano et al., 2021; Wang et al., 2018). The conflicting findings regarding DOX’s role in cardiac autophagy regulation highlight the need for further investigation.

The conflicting results regarding autophagy may be attributed to multiple factors, including variations in experiment models. Most methodologies for analyzing DIC, which employ models such as ion channel–overexpressing cells or animal models, frequently fail to fully and accurately replicate the effects of cardiac drugs in humans (Protze et al., 2019; McSweeney et al., 2019). This is due to interspecies differences in drug metabolism, cardiac structure and function. Recently, human induced pluripotent stem cells (hiPSCs) and human embryonic stem cells (hESCs) represent attractive cell sources for cell therapy and drug development (Cerneckis et al., 2024). hiPSC- and hESC-derived cardiomyocytes (hiPSC-CMs and hESC-CMs), which express key human cardiac ion channels and sarcomeric proteins, demonstrate contractile function, gene expression profiles, and electrophysiological phenotypes that closely resemble those of native human cardiomyocytes. Moreover, hiPSC-CMs and hESC-CMs offer a robust platform for large-scale drug screening and compound testing. hiPSC-CMs and hESC-CMs have been used to test drug-induced cardiac toxicity including DIC in recent works (Burridge et al., 2016; Maillet et al., 2016; Zhao and Zhang, 2017; Cui et al., 2019; Yang et al., 2022). However, the role and mechanisms of DIC in different CM models, particularly its multifaceted roles and mechanisms, including the dynamic changes and regulatory processes of autophagy, remain incompletely understood.

In the present study, we investigated the role and mechanisms of DIC using the hiPSC- and hESC-CM model system. Our results suggested DOX triggered apoptosis, ROS production and DNA damage in both hiPSC-CMs and hESC-CMs. Moreover, DOX enhanced autophagy via inhibiting mTOR signaling in hiPSC-CMs and hESC-CMs, ultimately leading to CM damage.

## 2 Materials and methods

### 2.1 Cardiomyocyte differentiation of hiPSCs and hESCs

The hiPSCs (American Type Culture Collection, ATCC) cell line present in this study were obtained from Stem Cell Bank of Chinese Academy of Sciences. The hESCs (H9 cell line, WA09, WiCell Research Institute) (Thomson et al., 1998) cell line present in this study were obtained from Shanghai Zhong Qiao Xin Zhou Biotechnology Co. Ltd. hiPSCs and hESCs were routinely cultured at 37 °C and 5% CO_2_ in mTeSR™ one complete maintenance medium (Stemcell Technologies, Inc.) on 6-well plates coated with Matrigel (BD Biosciences, Bedford, MA). Cells were passaged every 2–3 days using ACCUTASE™ cell detachment solution (Stemcell Technologies, Inc.). In order to direct differentiation into CMs as our previous work described (Ke et al., 2020), hiPSCs and hESCs were split at a 1:6 ratio and plated onto Matrigel-coated 12-well plates cultured for 3 days. When the hiPSCs and hESCs reached 85% confluence, 6 μM CHIR99021 (MedChemExpress) was added to RPMI/B27 without insulin medium (Gibco, Invitrogen, USA) to initiate the differentiation. On day 2, the medium was changed to RPMI/B27 minus insulin medium supplement with 3 μg/mL IWP2 (MedChemExpress). On day 5, the medium was changed to RPMI/B27 without insulin. On day 7, the differentiating cells were cultured in RPMI/B27 plus insulin medium (Gibco, Invitrogen, USA). On day 15, the cells were cultured with purification medium (Tohyama et al., 2013), which are comprised of glucose-depleted DMEM (Gibco, Invitrogen, USA), 213 μg/mL of l-ascorbic acid 2-phosphate supplemented (Sigma-Aldrich; Merck KGaA), and 500 μg/mL of recombinant human albumin (Oryzogen; Wuhan Healthgen Biotechnology, Corp.) combining with 4 mM L-lactic acid (Sigma-Aldrich; Merck KGaA). The medium was refreshed every 2 days during the purification process. On day 19, cells were maintained in RPMI/B27 plus insulin media. To investigate the effect of DOX (MedChemExpress) in hiPSC-CMs and hESC-CMs, cells at differentiation day 21 were dissociated by using 0.25% Trypsin-EDTA (Gibco, Invitrogen, USA) for 3 min at 37 °C and then centrifuged at 1,000 rpm for 5 min. Cells were replanted in DMEM (Gibco, Invitrogen, USA) containing 20% FBS (Gibco, Invitrogen, USA) for 24 h and changed in a chemically defined medium (CDM3) for maintenance. CDM3 medium consists of DMEM, 500 μg/mL of Oryza sativa–derived recombinant human albumin (Sigma-Aldrich; Merck KGaA), and 213 μg/mL of L-ascorbic acid 2-phosphate (Sigma-Aldrich; Merck KGaA).

### 2.2 Immunocytochemical staining

For immunocytochemical staining, we fixed cells with 4% PFA (Sigma), permeabilized them with 0.4% Triton X-100 (Sigma), and then incubated them with the primary antibody anti-cTnT (Abcam, USA, 1:400) and anti-Cx43 (Abcam, USA, 1:400) overnight at 4 °C. The nucleus was stained with DAPI (Invitrogen, USA). All samples were imaged with a Carl Zeiss microscope and processed with ZEN software.

### 2.3 Flow cytometry

For characterization of hiPSC-CMs and hESC-CMs, the differentiated CMs were collected after dissociation with 0.25% Trypsin-EDTA for 3 min. The cells were fixed and permeabilized with the Foxp3 Staining Buffer kit (Invitrogen, USA) for 30 min, followed by incubation with the primary antibody (anti-cTnT, Abcam, 1:100) for 1 h at room temperature. The PE-conjugated secondary antibody (Biolegend, 1:400) was added to the cells for 1 h at 4 °C. Apoptosis was assessed by flow cytometry, using an Annexin V-FITC Apoptosis Detection Kit (BD Pharmingen). The assay was conducted based on the instructions which were provided by the manufacturer. Briefly, after treatment with DOX, 5 × 10^5^ cells were rinsed twice in PBS and incubated in 100 μ L one x binding buffer. Five μ L of Annexin V-FITC was added to the cells and incubated at RT for 15 min in the dark. Propidium iodide was not added because of DOX autofluorescence which may interfere with its detection. All samples were analyzed with a flow cytometer (BD Accuri™ C6, BD Biosciences) and quantified with FlowJo software.

### 2.4 CCK8 assay

The viability of cells was evaluated by CCK8 assay (Beyotime Institute of Biotechnology, Jiangsu, China). Briefly, 1 × 10^4^ cells/well CM cells were seeded into 96-well plates (Gibco, Invitrogen, USA) and cultured for 3 days. The cells were then treated with the different doses (0, 0.1, 0.25, 0.5, 1, 1.5, 2.5, 5.0, 10, 25, 50, 100 μM) of DOX for 24 h. 24 h later, the medium was exchanged with a fresh medium containing CCK8 reagent and incubated for an additional 4 h at 37 °C. By using an enzyme-linked immunosorbent assay reader (Thermo Fisher Scientific, Inc.), the data were read at an absorbance of 450 nm wavelength.

### 2.5 LDH assay

Cells seeded on a 96-well culture plate were treated with DOX for 24 h. The LDH release assay was performed to detect cell cytotoxicity, following the manufacturer’s instructions (Beyotime Institute of Biotechnology, Jiangsu, China). Briefly, the culture medium was collected and incubated with the working mixture (lactate, INT solution and diaphorase) at 37 °C for 30 min in dark. The absorbance of the sample was measured by an enzyme-linked immunosorbent assay reader at 490 nm. First, cardiomyocytes were incubated with 5 mM 3-MA (MedChemExpress) (Hou et al., 2012) or 2.5 μM Rapamycin (MedChemExpress) (Zhou et al., 2007) for 12 h, then stimulated with 0.25 μM, 0.5 μM and 1 μM DOX, after that both were maintained for 24 h. The LDH release assay was conducted to detect cell cytotoxicity, following the manufacturer’s instructions as previously described.

### 2.6 Detection of reactive oxygen species (ROS)

Intracellular ROS generation was analyzed using a ROS Assay Kit (Beyotime Institute of Biotechnology, Jiangsu, China). In accordance with instructions, after treatment with different concentrations (0, 0.25, 0.5, 1 μM) of DOX for 24 h, cells were incubated with DCFH-DA for 30 min at 37 °C and then washed three times with the medium. The fluorescence intensity was measured using the fluorospectrophotometer at 488 nm excitation and 525 nm emission wavelengths (PerkinElmer, Canada). The ROS fluorescence intensity was normalized relative to the mean Hoechst fluorescence intensity. Meanwhile, the cells were detected by flow cytometry. For further analysis of mitochondrial ROS in hiPSC-CMs and hESC-CMs, the live cells were stained with MitoSOX Red dye (ThermoFisher, Life Technologies, USA) according to the manufacturer’s instructions. The images were obtained with Fluorescence microscopy, and analyzed with Image J software.

### 2.7 DNA damage

hiPSC-CMs or hESC-CMs were seeded on gelatin-coated coverslips and treated with or without DOX in 12-well plates. After 24 h, cells were washed twice with PBS, fixed in 4% formaldehyde in PBS at RT for 30 min, and then permeated with 0.1% Triton-100X at RT for 15 min. Next, the coverslips were washed, and blocked with 10% goat serum for 1 h at RT and then incubated with the first antibody (γ-H2AX, 1:200) overnight at 4 °C. The cells were then washed thrice for 5 min and stained with Alexa Fluor™ 555-conjugated antibody (1:500; Beyotime Institute of Biotechnology). After staining for 10 min with DAPI, cells were analyzed by an Optiphot-2 microscope (Nikon Corporation) equipped with a CCD video camera system (Optronics Engineering, Ltd.) or a confocal laser scanning microscopy (Optronics Engineering, Ltd.).

### 2.8 Analysis of autophagic flux

To analyze autophagic flux, hiPSC-CMs or hESC-CMs were replanted on Laser confocal dishes (JingAn Biological Technology Co., Ltd, Shanghai, China). Cells were washed twice with PBS and infected with adenovirus expressing mCherry-GFP-LC3B fusion protein (Ad-mCherry-GFP-LC3B; Beyotime Institute of Biotechnology, Jiangsu, China) at an MOI of 20 for 24 h. After infection, the cells were treated with 0.5 μM of DOX for 6, 12 and 24 h. Then, cells were fixed with 4% paraformaldehyde, and then visualized with a confocal laser scanning microscopy (Olympus). We measured autophagic flux at different time points by observing the color change of mCherry/GFP.

### 2.9 Transmission electron microscope

hiPSC-CMs or hESC-CMs prepared for TEM were treated with or without DOX for 24 h. The cells were harvested, fixed with 5% glutaraldehyde (Sigma-Aldrich; Merck KGaA) at 4 °C overnight, and then postfixed with 1% osmium tetroxide (Sigma-Aldrich; Merck KGaA) at RT for 1 h. After fixation, the cells were dehydrated through a graded series of ethanol (Sinopharm Chemical Reagent Co., Ltd). Subsequently, the samples were treated with Spurr embedding agents mixed with various specifications of acetone (Sigma-Aldrich; Merck KGaA). Then, the ultrathin sections were mounted on nickel grids, followed by staining with lead citrate, uranyl acetate and 50% ethanol solution (Sigma-Aldrich; Merck KGaA). Thin sections of each sample were observed and analyzed under a transmission electron microscope (Hitachi H −7,650).

### 2.10 Western blot analysis

Proteins were extracted from hiPSC-CMs or hESC-CMs with RIPA lysis buffer then centrifuged at 120,00× g for 30 min. The concentration was quantified with Bradford assay (Thermo-Fisher Biochemical Co. Ltd, Beijing, China). For immunoblotting analysis, 30 μg of total proteins were loaded into the PAGE-SDS gels. The proteins were separated and transferred to a nitrocellulose membrane (Millipore, Billerica, MA, USA). Next, the membrane was blocked in 5% Nonfat dry milk in PBST and then incubated overnight at 4 °C followed by primary antibodies against GAPDH (1:5000), mTOR (1:500), p-mTOR (1:500) and LC3B (1:1,000) purchased from Cell Signaling Technology. Subsequently, the membrane was rinsed with PBST and incubated with horseradish peroxidase-conjugated goat anti-rabbit or mouse immunoglobulin G (IgG) antibody (Proteintech) for 1 h at RT. Afterward, the membrane was then washed with PBST three times and visualized with enhanced chemiluminescence (ECL) reagent (Amersham; GE Healthcare Life Sciences), and images were captured with the ECL Tanon 5500 system (Tanon Science and Technology Co., Ltd.).

### 2.11 Statistical analysis

In all experiments, data were detected by three or four independent biological replicates (n = 3–4) and presented as means ± standard deviation (SD). Grayscale analysis was quantified using ImageJ software. Prior to parametric testing, the Shapiro–Wilk test confirmed normal distribution and the Brown–Forsythe test confirmed homogeneity of variances for all datasets. Data Statistical significance was analyzed by using the unpaired Student’s t-test or one-way ANOVA followed by Dunnett’s *post hoc* multiple comparison test. Two-way ANOVA was employed to analyze the effects of two independent variables. Differences were considered statistically significant when p < 0.05.

## 3 Results

### 3.1 Differentiation and characterization of hiPSC-CMs and hESC-CMs

**FIGURE 1:**
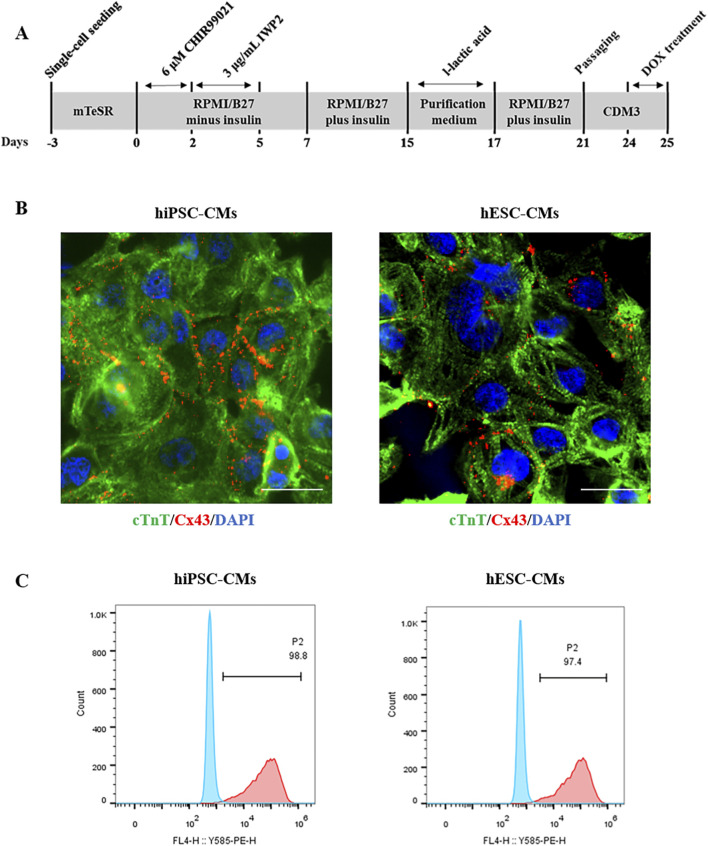
Differentiation and characterization of hiPSC-CMs and hESC-CMs. **(A)** Experimental scheme of differentiation protocol from hiPSCs and hESCs to differentiated cardiomyocytes. **(B)** Immunocytochemical staining of the cardiomyocyte marker cTnT in hiPSC-CMs and hESC-CMs at differentiation day 20. cTnT, green. CX43, red. DAPI, blue. Scar bar, 50 μm. **(C)** Flow cytometry analysis of the percentage of cTnT-positive (cTnT^+^) CMs at differentiation day 20.

hiPSCs and hESCs were differentiated to CMs as described previously (Ke et al., 2020) with slight modifications (Figure 1A). Immunocytochemical staining results showed that the cardiomyocyte-specific marker cTnT and the mature gap-junction marker Cx43 were expressed in hiPSC-CMs and hESC-CMs (Figure 1B). Flow cytometry analysis revealed that over 97% of the hiPSC-CMs and hESC-CMs were expressing the cTnT, indicating that these cells represent a highly pure cardiomyocyte population (Figure 1C). By day 10 post-differentiation, both hiPSC-CMs and hESC-CMs exhibited robust spontaneous contractions, providing qualitative evidence of electrophysiological activity (Supplementary Videos S1, S2).

### 3.2 DOX decreased the cell viability and induced cell apoptosis in hiPSC-CMs and hESC-CMs

**FIGURE 2:**
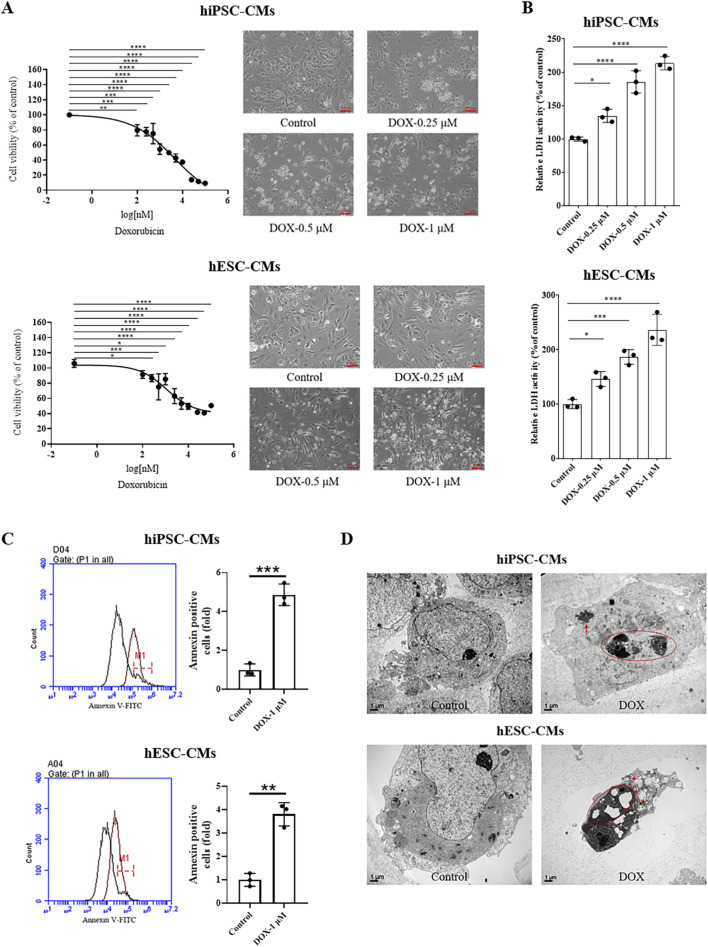
DOX decreased the cell viability and induced cell apoptosis of hiPSC-CMs and hESC-CMs. **(A)** hiPSC-CMs or hESC-CMs were treated with the different dose (0, 0.1, 0.25, 0.5, 1, 1.5, 2.5, 5.0, 10, 25, 50, 100 μM) DOX for 24 h. Cell viability was assessed by CCK-8 assay. Scale bar, 100 μm. P values were calculated using one-way ANOVA followed by Dunnett’s *post hoc* test comparing each DOX-treated group vs. the vehicle control group. *P < 0.05, **P < 0.01, ***P < 0.001, ****P < 0.0001. **(B)** The LDH activity of hiPSC-CMs and hESC-CMs after treatment with DOX (0, 0.25, 0.5, 1 μM) for 24 h. P values were calculated using one-way ANOVA followed by Dunnett’s *post hoc* test comparing each DOX-treated group vs. the vehicle control group. *P < 0.05, ***P < 0.001, ****P < 0.0001. **(C)** Following treatment with 1 μM DOX for 24 h, cells were collected and analyzed by flow cytometry using Annexin V-FITC staining. P values were calculated using an unpaired two-tailed t-test comparing the DOX-treated group vs. the vehicle control group. **P < 0.01, ***P < 0.001. **(D)** Morphologies of nuclear shrinkage and apoptotic bodies were observed by TEM in 1 μM DOX treatment group. The red circle represents nuclear shrinkage and the red arrow indicates autophagosome. Scale bar, 1 μm. (n = 3, mean = S.D).

In clinical studies, DOX can induce cardiomyocyte death within hours of intravenous administration in some patients at relatively low cumulative doses of 200–250 mg/m2 (Unverferth et al., 1983; Swain et al., 2003). The CCK8 results showed that DOX treatment resulted in a dose-dependent decrease in cell viability (Figure 2A). The cardiotoxicity was induced at a low concentration of DOX (0.1 μM for 24 h). We next investigated the cell damage caused by different concentrations of DOX (0–1 μM), which were chosen based on prior studies and are considered clinically relevant, as they fall well within the plasma concentration range observed in patients undergoing DOX therapy (Burridge et al., 2016; Frost et al., 2002). As shown in Figure 2B, LDH release significantly increased in a dose-dependent manner (0.25, 0.5 and 1 μM) following DOX treatment. Subsequently, we investigated the DOX-induced apoptosis in hiPSC-CMs and hESC-CMs. At a high concentration of 1 μM, DOX caused the most significant cell damage, prompting its use in the present study for apoptosis analysis. Flow cytometry analysis demonstrated that exposure of CMs to 1 μM DOX for 24 h resulted in a significant increase of Annexin V-FITC^+^ cells (an indicator of total apoptotic cells) compared to vehicle-treated control cells (Figure 2C). Moreover, morphologies of nuclear shrinkage and apoptotic bodies, indicative of typical apoptotic features, were observed by TEM in the DOX-treated group (Figure 2D). These data demonstrate that DOX treatment decreases cell viability and induces apoptosis in hiPSC-CMs and hESC-CMs. Collectively, these results from our study align with previous cellular and human biopsy reports (Burridge et al., 2016; Maillet et al., 2016; Zhao and Zhang, 2017; Cui et al., 2019), confirming the cardiotoxic effects of DOX and establishing a reliable *in vitro* model for assessing DIC in hiPSC-CMs and hESC-CMs.

### 3.3 DOX increased cellular ROS production and triggered DNA damage of hiPSC-CMs and hESC-CMs

**FIGURE 3:**
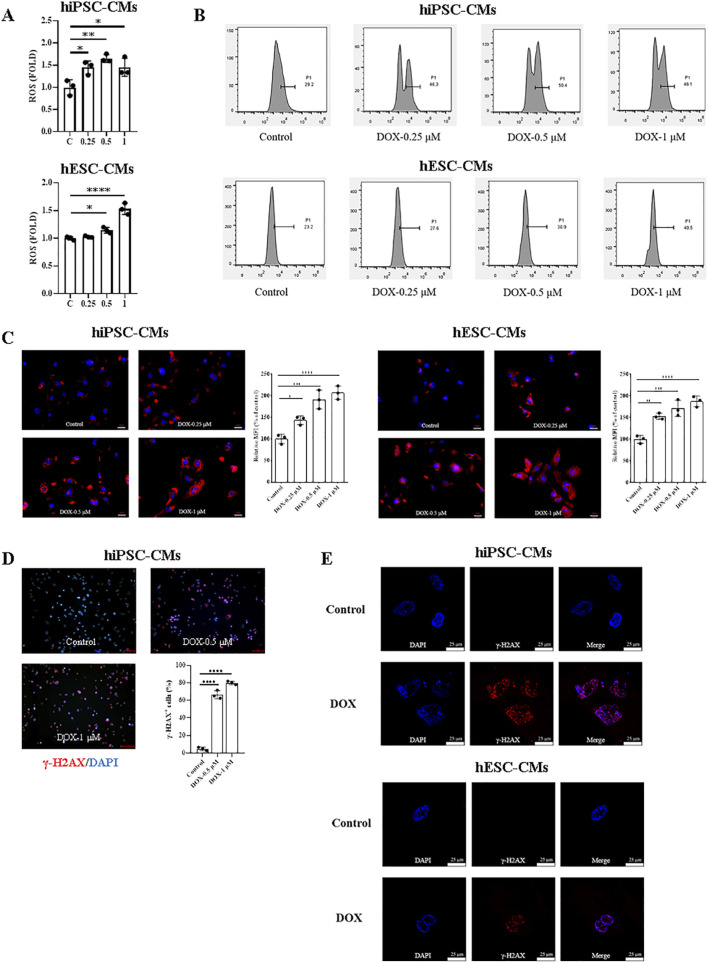
ROS production and DNA damage induced by DOX in hiPSC-CMs and hESC-CMs. **(A)** Exposure of hiPSC-CMs or hESC-CMs to different dose (0, 0.25, 0.5, 1 μM) DOX for 24 h resulted in a significant increase in ROS production as measured by the DCFH-DA probe. P values were calculated using one-way ANOVA followed by Dunnett’s *post hoc* test comparing each DOX-treated group vs. the vehicle control group. *P < 0.05, **P < 0.01, ****P < 0.0001. **(B)** The representative data of flow cytometry assessed by DCFH-DA in hiPSC-CMs and hESC-CMs with or without DOX treatment. **(C)** Mitochondrial ROS were quantified as relative median fluorescence intensity (MFI) of MitoSOX normalized to vehicle control in hiPSC-CMs and hESC-CMs treated overnight with 0.25, 0.5 and 1 μM DOX. MitoSOX is shown in red, and DAPI nuclear staining is shown in blue. Scale bars, 50 μm. Data were analyzed by one-way ANOVA with Dunnett’s post-test; *P < 0.05, **P < 0.01, ***P < 0.001, ****P < 0.0001 *versus* control. **(D)** Representative fluorescence images and quantitative data of DNA double-stranded break in hiPSC-CMs treated overnight with 0.5 and 1 μM DOX. γ-H2AX staining is shown in red, and DAPI nuclear staining is shown in blue. Scale bars, 100 μm. Data were analyzed by one-way ANOVA with Dunnett’s post-test; ****P < 0.0001 *versus* control. **(E)** Representative fluorescence images of DNA double-stranded break in hiPSC-CMs and hESC-CMs treated overnight with 0.5 μM DOX. γ-H2AX, red; DAPI, blue. Scale bars, 25 μm. (n = 3, mean = S.D).

To investigate the damage role of DOX in hiPSC-CMs and hESC-CMs, we first measured intracellular ROS production following DOX treatment using the probe DCFH-DA. As shown in Figure 3A, compared with the control, DOX induced a significant increase in intracellular ROS in hiPSC-CMs at 0.25, 0.5, and 1 μM, whereas in hESC-CMs significance was observed only at 0.5 and 1 μM. Similarly, the results were confirmed by flow cytometry analysis (Figure 3B). Additionally, a dose-dependent increase in MitoSOX red fluorescence was observed in cells exposed to DOX (Figure 3C). Next, the level of double-stranded DNA damage was assessed through staining for phosphorylated H2A histone family member X (γ-H2AX) on serine 139. As shown in Figure 3D, DOX treatment induced γ-H2AX expression in hiPSC-CMs in a dose-dependent manner, with significant DNA damage evident at a concentration of 0.5 μM. Therefore, we evaluated γ-H2AX expression in hiPSC-CMs and hESC-CMs treated with 0.5 μM DOX using laser confocal microscopy. The results demonstrated that DOX induced an increase in γ-H2AX expression in both hiPSC-CMs and hESC-CMs groups (Figure 3E). These findings indicate that DOX increases ROS levels and triggers DNA damage, leading to apoptosis, which represents one of the mechanisms of DIC.

### 3.4 DOX induced autophagy of hiPSC-CMs and hESC-CMs

**FIGURE 4:**
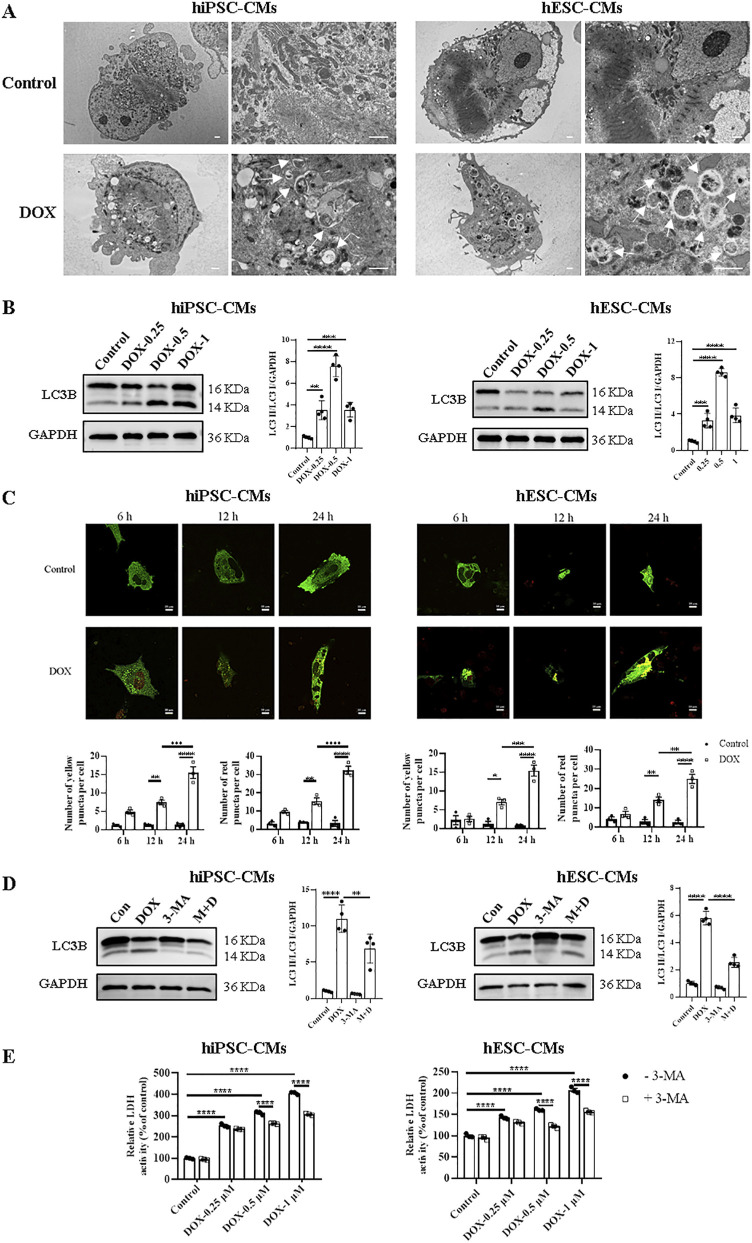
Treatment with DOX upregulated hiPSC-CMs and hESC-CMs autophagy. **(A)** Representative TEM images of hiPSC-CMs and hESC-CMs treated overnight with 0.5 μM DOX. Arrowhead, autophagosomes and autolysosomes. Scale bars, 1 μm. **(B)** Western blot analysis of LC3B in hiPSC-CMs and hESC-CMs following DOX (0, 0.25, 0.5, 1 μM) treatment. P values were calculated using one-way ANOVA followed by Dunnett’s *post hoc* test comparing each DOX-treated group vs. the vehicle control group. ***P < 0.001, ****P < 0.0001. **(C)** Representative fluorescence images o and quantitative data f hiPSC-CMs and hESC-CMs expressing mCherry-GFP-LC3 and treated with 0.5 μM DOX for 6, 12 and 24 h, respectively. Autophagosomes, yellow puncta; autolysosome, red puncta. Scale bars, 10 μm. Data were analyzed by two-way ANOVA; *P < 0.05, **P < 0.01, ***P < 0.001, ****P < 0.0001. **(D,E)** hiPSC-CMs and hESC-CMs were incubated with 5 mM 3-MA for 12 h prior to stimulation with 0.5 μM DOX or different concentrations of DOX (0, 0.25, 0.5, 1 μM), then both were maintained for 24 h. **(D)** The effect of 3-MA on the expression ratio of LC3 II/LC3 I in hiPSC-CMs and hESC-CMs analyzed by Western blotting. P values were calculated using one-way ANOVA followed by Dunnett’s *post hoc* test for multiple comparisons among all groups (Control, DOX, 3-MA, M + D). **P < 0.01, ****P < 0.0001. **(E)** By spectrophotometry, LDH activity in the culture media was measured. P values were calculated using two-way ANOVA. ****P < 0.0001. The lower band is LC3 II and the higher one is LC3 I on LC3B membrane. GAPDH was used as a loading control and grayscale analysis was performed for statistics. (n = 3-4, mean = S.D.).

Autophagy has dual functions in both physiology and pathology. To evaluate the changes in autophagy, we first observed ultrastructure changes and autophagosome formation in hiPSC-CMs and hESC-CMs by TEM. The results revealed an accumulation of autophagosomes and autolysosomes in DOX-treated groups (Figure 4A). Western blots were performed to detect the changes in the ratios of LC3 II/LC3 I in hiPSC-CMs and hESC-CMs treated with or without DOX. We found that DOX significantly upregulated the ratio of LC3 II/LC3 I in the hiPSC-CMs and hESC-CMs (Figure 4B). The most pronounced changes in autophagy-related proteins were noted at a DOX concentration of 0.5 μM. Therefore, the concentration of 0.5 μM was utilized in subsequent studies to investigate the effects of DOX on autophagy and its underlying mechanisms. To further study DOX-induced autophagic flux, we detected autophagy in hiPSC-CMs and hESC-CMs infected with adenovirus expressing a dual-fluorescence reporter mCherry-GFP-LC3B fusion protein (Ad-mCherry-GFP-LC3B) at a different time point (6 h, 12 h, and 24 h). Treatment with DOX resulted in an increase of autophagic flux within 6 h, as indicated by the number of both yellow and red puncta, with the maximum effect observed at 24 h (Figure 4C). Moreover, the DOX-induced increases in the LC3 II/LC3 I ratio were attenuated by 3-MA, an autophagy inhibitor, as shown in Figure 4D. In addition, to assess the role of autophagy in DOX-induced cardiomyocyte death, we evaluated the effects of 3-MA on LDH release. In Figure 4E, compared with the control, DOX induced an increase in LDH level, indicating cellular damage. However, pretreatment with the autophagy inhibitor 3-MA led to a significant yet partial reduction in DOX-induced LDH release at both 0.5 and 1 μM, resulting in a partial rescue effect (Figure 4E; Supplementary Table S1). Collectively, these results suggest that DOX upregulates autophagy in both hiPSC-CMs and hESC-CMs, which partially contribute to cardiotoxicity.

### 3.5 DOX inhibited mTOR signaling in hiPSC-CMs and hESC-CMs

**FIGURE 5:**
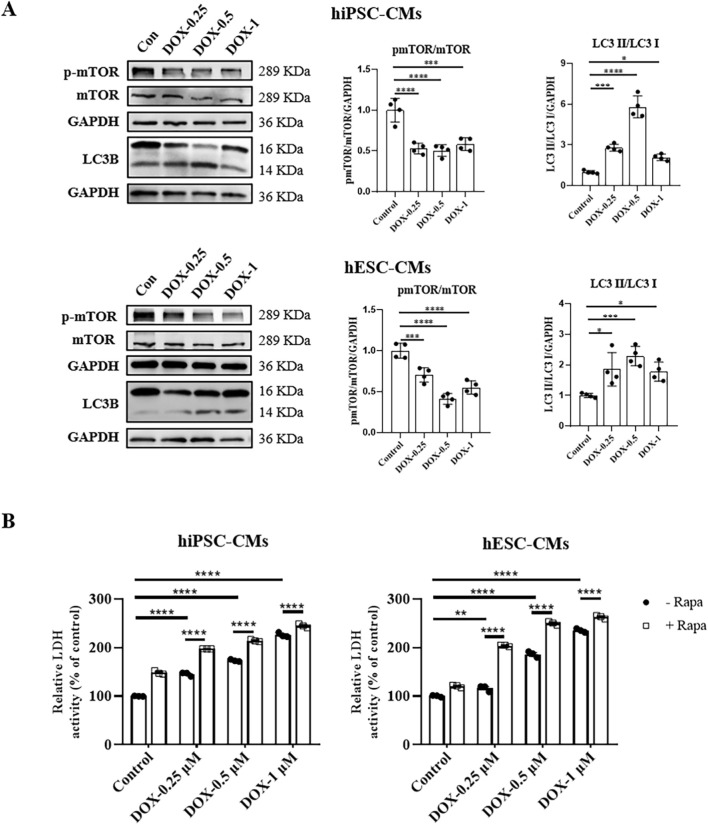
DOX inhibited mTOR signaling in hiPSC-CMs and hESC-CMs. **(A)** Western blot analysis of mTOR, p-mTOR, and LC3 II/LC3 I in hiPSC-CMs and hESC-CMs following DOX (0, 0.25, 0.5, 1 μM) treatment. P values were calculated using one-way ANOVA followed by Dunnett’s *post hoc* test comparing each DOX-treated group vs. the vehicle control group. *P < 0.05, ***P < 0.001, ****P < 0.0001. **(B)** hiPSC-CMs or hESC-CMs were treated with 2.5 μM rapamycin for 24 h. Then CMs were exposed to different concentrations of DOX (0, 0.25, 0.5, 1 μM) for 24 h. In the culture media, LDH activity was assessed by spectrophotometry. P values were calculated using two-way ANOVA. **P < 0.01, ****P < 0.0001. The lower band is LC3 II and the higher band is LC3 I on LC3B membrane. GAPDH was used as a loading control and grayscale analysis was performed for statistics. (n = 3-4, mean = S.D).

The kinase mTOR functions as a key signaling ‘station’ in the regulation of cellular metabolism, promoting protein synthesis while inhibiting the induction of autophagy (Liang, 2010). Although other pathways such as PI3K/AKT and AMPK also play roles in autophagy regulation, mTOR is widely recognized as one of the most pivotal and extensively studied mediators of autophagic processes (Shackebaei et al., 2024). Given the gatekeeper role of mTOR in autophagy regulation, we investigated whether DOX alters autophagy in hiPSC-CMs and hESC-CMs through mTOR signaling. We found that DOX treatment notably decreased mTOR activation in hiPSC-CMs and hESC-CMs, as evidenced by the reduced phosphorylation at Ser-2248 across various concentrations (Figure 5A). To further confirm the role of mTOR signaling in autophagy induced by DOX, we next treated hiPSC-CMs and hESC-CMs with rapamycin (RAPA), an mTOR inhibitor. In hiPSC-CMs, RAPA alone increased LDH release and exacerbated DOX-induced cell death, although without significant additive cytotoxicity (Figure 5B; Supplementary Table S1). In contrast, in hESC-CMs co-treatment with RAPA and DOX (0.25 and 0.5 μM) resulted in a significant additive increase in LDH release, suggesting the inhibition of mTOR enhances the cytotoxic effects of DOX (Figure 5B; Supplementary Table S1). Collectively, these experiments demonstrate that DOX stimulates autophagy in hiPSC-CMs and hESC-CMs through inhibiting mTOR signaling.

## 4 Discussion

DOX is one of the most effective anthracycline chemotherapy agents for treating a wide range of malignancies but causes cardiotoxicity in many patients (Jones and Dass, 2022). In the present study, our investigation focused on the features and mechanisms of DIC using the hiPSC- and hESC-CM model. Our results suggested that DOX treatment decreased cell viability and induced cell apoptosis via the accumulation of ROS and DNA damage. Notably, our study further investigated the function of autophagy in DIC and demonstrated that DOX promoted autophagy of hiPSC-CMs and hESC-CMs by inhibiting mTOR signaling.

The mechanism and pathogenesis of DIC remain controversial and obscure, despite extensive research over the last half-century. Animal and heterologous cell models help clarify DIC mechanisms but may not fully capture human cardiac complexities. hiPSC-CMs and hESC-CMs provide a human-based, high-throughput, and genetically diverse platform that can significantly enhance our understanding of DIC (Burridge et al., 2016; Maillet et al., 2016; Zhao and Zhang, 2017; Cui et al., 2019; Yang et al., 2022). In our research, we first utilized both hiPSC-CM and hESC-CM models to investigate the multifaceted effects and potential mechanisms of DIC, with a specific focus on the dynamic changes and regulatory processes associated with autophagy.

A commonly cited pathway involves the DOX-induced generation of ROS associated with mitochondrial dysfunction (Songbo et al., 2019; Yarmohammadi et al., 2021). Our data showed that DOX increased ROS production of hiPSC-CMs and hESC-CMs, in agreement with previous reports (Songbo et al., 2019). The generation of ROS by redox cycling causes DNA damage, an early event in DIC, which was also confirmed in our data (L'Ecuyer et al., 2006). DOX significantly increased ROS levels in both the intracellular and mitochondria of hiPSC-CMs and hESC-CMs; however, the magnitude and dose-response dynamics differed between the two models (Supplementary Table S1), highlighting the necessity for patient-specific or lineage-specific mechanistic profiling of oxidative stress responses. Moreover, mitochondrial ROS exhibited a greater increase compared to intracellular ROS (Supplementary Table S1), consistent with mitochondria representing the primary source of ROS generation in DIC (Songbo et al., 2019). DNA damage, one of the most significant effects induced by chemical agents, triggers different stress responses in various cell models, either repairing the damage or leading to cell death (Naselli et al., 2024). In our study, DOX induces DNA damage in hiPSC-CMs and hESC-CMs, which subsequently results in their apoptosis. In addition, DIC mediated by topoisomerase-IIβ causes transcriptional modulation of nuclear and mitochondrial genes and DNA-damage-induced apoptosis (Zhang et al., 2012). The ROS level was increased in H9c2 cells after treatment with DOX which promotes the NLRP3 inflammasome activation and secretion of IL-1β (Wei et al., 2020). The role of NLRP3 inflammasome in our cell model needs to be further evaluated.

Autophagy has dual functions, enhancing cellular survival by degrading damaged or obsoleted proteins and organelles under physiological conditions or inducing cell death under pathological conditions (Marshall and Vierstra, 2018). There are many steps involved in autophagy, as it comprises multiple steps such as membrane nucleation, elongation, and completion of the autophagosome, autophagosome fusion with the lysosome to form autolysosome, and autolysosomal degradation (Mizushima, 2007). LC3, an autophagy marker, plays a crucial role in autophagosome biogenesis and maturation (Mizushima, 2020). LC3 II is formed by LC3 I conjugated to phosphatidylethanolamine, which amount is related to the number of autophagosomes (Mizushima and Yoshimori, 2007). Advanced imaging and marker-based techniques have proven to be invaluable tools for precisely characterizing and deeply understanding the dual roles of autophagy in cellular stress and cardiotoxicity (Naselli et al., 2024; Klionsky et al., 2021). In our research, Western blot analysis demonstrated that DOX significantly upregulated the LC3 II/LC3 I ratio both hiPSC-CMs and hESC-CMs, with the maximal increase consistently observed at 0.5 μM (Supplementary Table S1). Furthermore, utilizing advanced imaging and marker-based techniques, TEM visualization revealed a significant increase in autophagosome formation in DOX-treated groups, and the results of autophagy flux assays using Ad-mCherry-GFP-LC3B indicated a time-dependent accumulation of autophagosomes. Thereby DOX can be considered to stimulate autophagy in hiPSC-CMs and hESC-CMs via increasing the number of autophagosomes. Although DOX enhanced autophagy in both hiPSC-CMs and hESC-CMs, static snapshots may bias quantification; higher-throughput and dynamic analyses are further required to accurately assess potential differences. Recently, some studies suggested that DOX treatment increased the accumulation of autophagosomes in response to the autophagic degradation process inhibition, such as impairing lysosomal function as well as autophagosome/autolysosome fusion, but has little effect on autophagosome formation *in vivo* (mice) and *in vitro* (NRCM) (Li et al., 2016; Abdullah et al., 2019). We did not directly assess lysosomal activity or the fusion of autophagosomes with autolysosomes using LysoTracker or chloroquine-based assays; thus, the possibility that DOX may impair autophagic degradation remains insufficiently investigated and warrants further research. Autophagy functions as a double-edged sword: on one hand, it protects cells by degrading and recycling damaged organelles and proteins; on the other hand, excessive autophagy can induce cell death. In our work, DOX-induced hyperactive autophagy exacerbated cellular damage, ultimately leading to cell death in hiPSC-CMs and hESC-CMs. Furthermore, we found that inhibition of autophagy formation induced by DOX using 3-MA, an autophagy inhibitor downregulating PI3 kinase complex, decreased expression of LC3 II/LC3 I and improved cell viability of hiPSC-CMs and hESC-CMs. Nevertheless, 3-MA only partially alleviated the injury, and cell viability remained below baseline levels. This indicates that autophagy may represent one aspect of the injury mechanism in DIC. The biological meaning of this effect and the precise contribution of autophagy to overall cell death remain to be fully elucidated. Our findings underscore the intricate and pivotal role of autophagy in DIC and highlight the therapeutic potential of modulating autophagy. However, we did not dissect the downstream of autophagy flux mediated by DOX. Moreover, previous studies have demonstrated that dox-induced activation of autophagy is likely pathological and contributes to cellular dysfunction and apoptosis (Dirks-Naylor, 2013). In our study, DOX at 0.5 μM induces peak autophagy and concurrently increases cell death in hiPSC-CMs and hESC-CMs, but apoptosis was not assessed (Supplementary Table S1). At 1 μM, DOX-induced autophagy remains elevated compared to controls but declines, while apoptosis markedly increases, with distinct fold changes in hiPSC-CMs *versus* hESC-CMs (Supplementary Table S1). A study reported that a high concentration of DOX (1 μM) was associated with DNA damage, PARP-1 dissociation, and severe apoptosis in mouse stem cell-derived cardiomyocytes (Cunha-Oliveira et al., 2018). Whether the apoptotic surge is caused by concurrent autophagic activation or occurs independently remains unclear. Future studies should combine apoptosis detection methods and inhibitors like caspase inhibitors to analyze the temporal dynamics of autophagic flux and apoptosis in DIC, clarify their interaction, and identify key mechanisms.

What mechanisms are involved in the stimulation of cardiac autophagy by DOX? The mTOR protein, an atypical serine/threonine kinase, exerts as a critical signaling ‘station’ in regulating cell homeostasis and stress responses, which stimulates protein synthesis and suppresses the induction of autophagy (Liang, 2010; Liu et al., 2024; Guseva et al., 2024). mTOR interacts with the ULK1-Atg13-FIP200 complex, which is essential for the onset of autophagosome formation in mammals, and phosphorylates ULK1 and Atg13 to inhibit autophagy (Ganley et al., 2009). It has been demonstrated that mTOR signaling is essential for heart physiological processes regulation such as growth, aging, and lifespan, as well as for playing a pivotal role in pathological conditions such as atherosclerosis and ischemia–reperfusion injury (Park et al., 2016; Yuan et al., 2014; Li et al., 2019; Gurusamy et al., 2010; Liu et al., 2021). Several studies have explored the impact of DOX on the mTOR signaling pathway (Yarmohammadi et al., 2023). However, the role and mechanisms of mTOR in DIC remain inconsistent and inconclusive (Shackebaei et al., 2024). Some studies have reported a reduction in mTOR protein activation within cardiac tissue, while others have observed an increase in activation (Yarmohammadi et al., 2023). Our findings indicate that following DOX treatment, p-mTOR levels decreased significantly, suggesting that DOX may induce autophagy by inhibiting mTOR signaling. RAPA, an mTOR inhibitor, further reduced cell viability, particularly at 0.25 µM DOX in hESC-CMs. hESC-CMs and hiPSC-CMs exhibit distinct responses to RAPA-induced injury. This difference may be attributed to DOX-mediated suppression of p-mTOR to a low baseline level in hiPSC-CMs, thereby limiting the potential for additional inhibitory effects by RAPA. Alternatively, the underlying injury mechanisms may differ between the 2 cell types. These findings highlight the need for further investigation to clarify the biological significance of these observations. This suppression of mTOR signaling may occur via several classical upstream regulatory pathways, including the activation of AMPK and the inhibition of the PI3K/AKT pathway (Shackebaei et al., 2025). DOX has been demonstrated to activate AMPK in both H9C2 cardiomyocytes and mouse hearts (Chen et al., 2011; Pointon et al., 2010). Furthermore, downregulation or disruption of the PI3K/AKT/mTOR signaling pathway is increasingly recognized as a critical mechanism in DIC (Yu et al., 2020; Zhang et al., 2020). Recently, a study utilizing GEO transcriptomic data and animal model validation has identified a 23-gene autophagy signature—including Akt1, Hif1a, and Mapk3—that highlights potential mechanistic and therapeutic targets (Wu et al., 2024). However, current understanding of the mTOR axis predominantly using cell lines or acute high-dose animal models, resulting in a significant gap in clinically relevant contexts. In this study, we demonstrate the essential role of mTOR in DIC using hiPSC-CMs and hESC-CMs. In future studies, we will use this model with multi-omics profiling—including transcriptomics, proteomics, and metabolomics—to develop precision mTOR-targeted therapies for DIC.

Li et al. reported that DOX blocked cardiomyocyte autophagic flux by altering lysosomal function in mice, independent of mTOR activation (Li et al., 2016). We hypothesize that DOX-induced activation of autophagosome synthesis promotes autophagy damage in CMs. It is plausible that the conclusions may come from differences in the cell models, the dose and time of DOX treatment, and so on. Recent studies have identified novel regulated cell death pathways and elucidated their involvement in the pathogenesis of DIC, including ferroptosis and pyroptosis. Ferroptosis is an iron-dependent form of cell death characterized by uncontrolled lipid peroxidation, which ultimately leads to membrane rupture (Ru et al., 2024). Lipid peroxides represent one of the major sources of ROS involved in DIC, and the role of iron in this process has been well established (Christidi and Brunham, 2021). Our study revealed a significant elevation in ROS levels in hiPSC-CMs and hESC-CMs exposed to DIC; however, the precise origin of this oxidative stress and its mechanistic link to ferroptosis remain to be fully elucidated. Pyroptosis is a novel form of programmed cell death that is mediated by caspase-1 activation and involves the release of substantial pro-inflammatory mediators (Bai et al., 2025). Evidence increasingly shows that DOX induces excessive autophagy through GSDMD or miR-34a-5p upregulation, promoting pyroptosis in mouse cardiomyocytes and contributing to cardiac toxicity (Qu et al., 2022; Zhong et al., 2023). In future studies, we aim to investigate the mechanistic crosstalk between DOX-induced autophagy and pyroptosis in hiPSC-CMs and hESC-CMs.

In conclusion, our work demonstrated DOX-induced cardiotoxicity via ROS production, DNA damage, apoptosis, and autophagy in hiPSC-CMs and hESC-CMs. Moreover, the present work suggested that DOX enhanced autophagy via inhibiting mTOR signaling in hiPSC-CMs and hESC-CMs. Overall, these data provide a better understanding of DIC and indicate that the mTOR signaling pathway and its modulation of autophagy represent a valuable therapeutic target of DIC.

## Data Availability

The original contributions presented in the study are included in the article/Supplementary Material, further inquiries can be directed to the corresponding authors.
