# Supplementary material for: Doxorubicin induces cardiotoxicity by enhancing autophagy via mTOR signaling in hiPSC- and hESC-derived cardiomyocytes
Source: Front Cell Dev Biol. 2025 Nov 25;13:1616235. doi: 10.3389/fcell.2025.1616235 (PMC12685840; doi:10.3389/fcell.2025.1616235)
Supplement: Supplementary file 3 [file DataSheet1.docx]

**Supplementary Files**

**
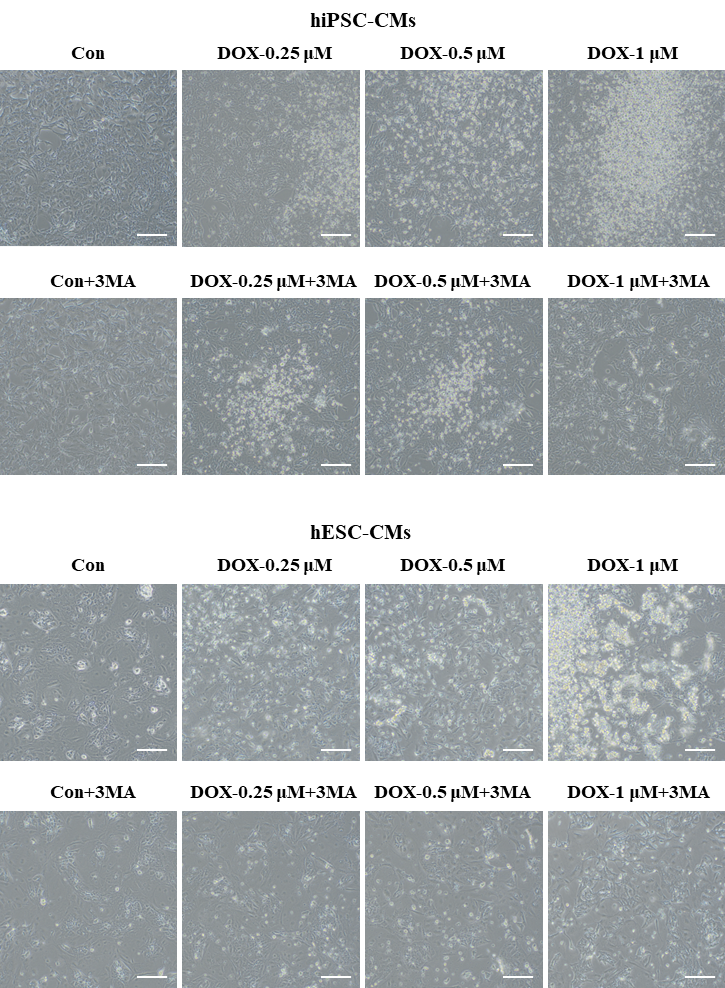
**

**Supplementary Figure 1.** **Representative cellular morphology was assessed by microscopy after pre-treatment with 3-MA (12 h) followed by exposure to DOX (0, 0.25, 0.5 or 1 μM) for 24 h; scale bar = 200 μm.**

**
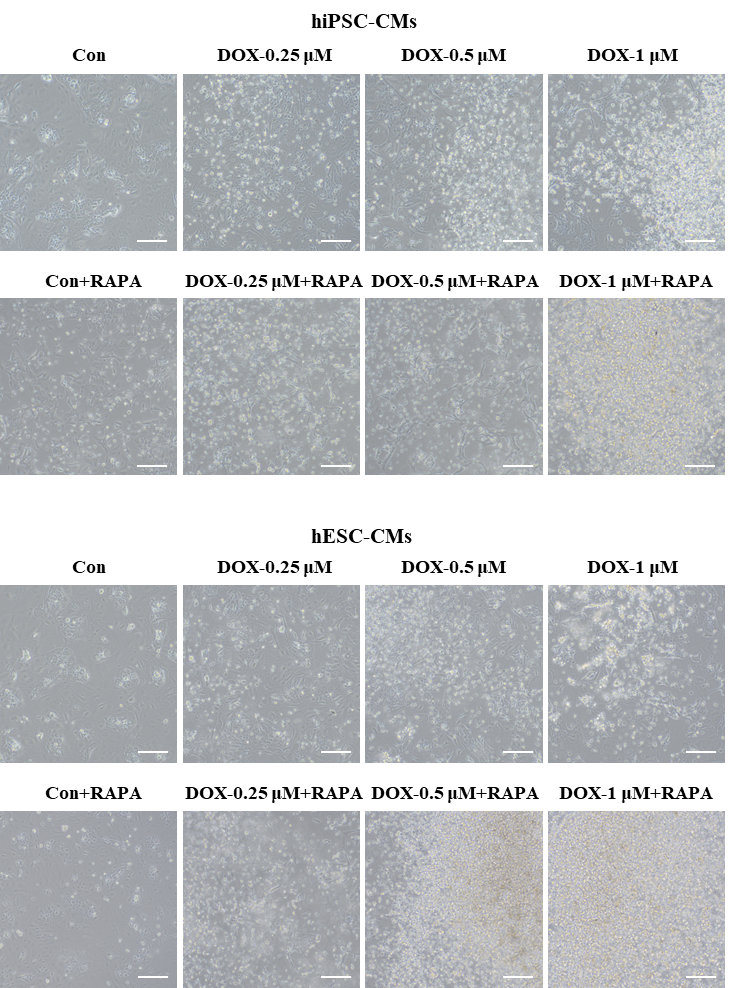
**

**Supplementary Figure 2.** **Representative cellular morphology was assessed by microscopy after pre-treatment with RAPA (12 h) followed by exposure to DOX (0, 0.25, 0.5 or 1 μM) for 24 h; scale bar = 200 μm.**

**Supplementary table 1. effects of DOX on hiPSC-CMs and hESC-CMs: percentage change in cell death, apoptosis, intracellular/mitochondrial ROS, and autophagy**

| **Cell type** | **DOX (µM)** | **Time** | **Cell death (LDH)** | **Intracellular ROS (DCFH-DA)** | **Mitochondrial ROS (MitoSOX)** | **Apoptosis (Annexin V⁺)** | **Autophagy (LC3B-II/I)** | **Cell death (LDH)** | | **Cell death (LDH)** | |
| --- | --- | --- | --- | --- | --- | --- | --- | --- | --- | --- | --- |
|  |  |  |  |  |  |  |  | -3MA | +3MA | -Rapamycin | +Rapamycin |
| **hiPSC-CMs** | Control | 24 h | 100% ± 3% | 100% ± 17% | 100% ± 11% | 100% ± 30% | 100% ± 12% | 100% ± 3% | 95% ± 3% | 100% ± 1% | 149% ± 4% |
|  | DOX-0.25 | 24 h | 135% ± 10% | 144% ± 15% | 143% ± 10% | / | 352% ± 88% | 253% ± 8% | 237% ± 2% | 145% ± 4% | 198% ± 0.1% |
|  | DOX -0.5 | 24 h | 186% ± 17% | 165% ± 9% | 191% ± 22% | / | 756% ± 95% | 312% ± 9% | 263% ± 3% | 173% ± 2% | 215% ± 2% |
|  | DOX -1 | 24 h | 214% ± 10% | 145% ± 20% | 207% ± 16% | 486% ± 55% | 355% ± 68% | 406% ± 6% | 306% ± 5% | 226% ± 5% | 245% ± 4% |
| **hESC-CMs** | Control | 24 h | 100% ± 8% | 100% ± 3% | 100% ± 9% | 100% ± 28% | 100% ± 15% | 100% ± 4% | 95% ± 4% | 100% ± 2% | 120% ± 3% |
|  | DOX-0.25 | 24 h | 146% ± 14% | 103% ± 2% | 153% ± 7% | / | 327% ± 81% | 142% ± 4% | 131% ± 3% | 116% ± 6% | 204% ± 2% |
|  | DOX-0.5 | 24 h | 187% ± 13% | 115% ± 5% | 172% ± 19% | / | 859% ± 43% | 161% ± 3% | 122% ± 4% | 186% ± 6% | 250% ± 4% |
|  | DOX-1 | 24 h | 236% ± 28% | 153% ± 10% | 188% ± 12% | 380% ± 50% | 387% ± 81% | 207% ± 7% | 156% ± 3% | 235% ± 4% | 263% ± 4% |
